# An Artificial Neural Network to Eliminate the Detrimental Spectral Shift on Mid-Infrared Gas Spectroscopy

**DOI:** 10.3390/s23198232

**Published:** 2023-10-03

**Authors:** Sanghoon Chin, Jérôme Van Zaen, Séverine Denis, Enric Muntané, Stephan Schröder, Hans Martin, Laurent Balet, Steve Lecomte

**Affiliations:** 1 Centre Suisse d’Electronique et de Microtechnique SA (CSEM), CH-2002 Neuchâtel, Switzerland; jerome.vanzaen@csem.ch (J.V.Z.); severine.denis@csem.ch (S.D.); enric.muntanecalvo@csem.ch (E.M.); laurent.balet@csem.ch (L.B.); steve.lecomte@csem.ch (S.L.); 2 SenseAir AB, 82060 Delsbo, Sweden; stephan.schroder@senseair.com (S.S.); hans.martin@senseair.com (H.M.)

**Keywords:** spectral analysis, artificial neural network, quantitative gas analysis, trace gas sensing, mid-infrared, absorption spectroscopy, supercontinuum source

## Abstract

We demonstrate the successful implementation of an artificial neural network (ANN) to eliminate detrimental spectral shifts imposed in the measurement of laser absorption spectrometers (LASs). Since LASs rely on the analysis of the spectral characteristics of biological and chemical molecules, their accuracy and precision is especially prone to the presence of unwanted spectral shift in the measured molecular absorption spectrum over the reference spectrum. In this paper, an ANN was applied to a scanning grating-based mid-infrared trace gas sensing system, which suffers from temperature-induced spectral shifts. Using the HITRAN database, we generated synthetic gas absorbance spectra with random spectral shifts for training and validation. The ANN was trained with these synthetic spectra to identify the occurrence of spectral shifts. Our experimental verification unambiguously proves that such an ANN can be an excellent tool to accurately retrieve the gas concentration from imprecise or distorted spectra of gas absorption. Due to the global shift of the measured gas absorption spectrum, the accuracy of the retrieved gas concentration using a typical least-mean-squares fitting algorithm was considerably degraded by 40.3%. However, when the gas concentration of the same measurement dataset was predicted by the proposed multilayer perceptron network, the sensing accuracy significantly improved by reducing the error to less than ±1% while preserving the sensing sensitivity.

## 1. Introduction

Photonic sensing systems in the mid-infrared (MIR) wavelength range have recently attracted substantial attention from the optical community due to their outstanding capability to detect minute traces of molecules in complex gas mixtures. This is possible because the fingerprint of rotational and vibrational resonances of gas molecules originating from biological and chemical activities along this spectral window is a few orders of magnitude stronger than other spectral ranges. Therefore, MIR spectroscopy systems have been extensively employed for various applications, such as air quality monitoring, health diagnostics, and scientific research [1,2,3,4,5,6]. In this context, the generation of MIR light sources emitting in the wavelength range of 2–10 µm has been effectively demonstrated using various techniques, such as quantum cascade lasers [7,8], optical parametric oscillators [9,10], and supercontinuum lasers [11,12,13,14]. Among them, the high-brightness supercontinuum light source, associated with a high-resolution diffractive grating spectrometer, shows high performance in the accurate detection of minute traces of multispecies gas molecules [5].

However, we observed that any presence of thermal fluctuation of the gaseous analyte can lead to detrimental thermally induced mechanical stresses to optical components in the system, causing beam steering. Therefore, unwanted spectral shifts may occur in the measured absorption spectrum, which, in turn, results in a non-negligible amount of error for the gas concentration computation, traditionally performed by the least-squares fitting process between the measured spectrum and the database reference spectrum [11]. Such a spectral calibration issue has already been addressed in a similar grating-based gas sensing system [14] and has been solved by cross-correlation calculation between the measured absorbance spectrum and the reference spectrum to estimate the exact amount of the spectral shift caused by mechanical disturbances in the system. Yet, this solution requires a heavy mathematical computation that slows down the measurement speed and the clear presence of known gas species to precisely compare with the reference, which might limit the sensing performance for ambient air quality monitoring.

Over the last decade, ANNs have been applied to multiple scientific research domains and engineering applications due to their relevance for complex nonlinear problems. For instance, the intrinsic cross-sensitivity of multiple physical parameters and harmful nonlinear effects in a sensing system could be precisely resolved thanks to a properly trained machine learning algorithm [15,16,17,18]. Even a deep learning-based machine learning algorithm can significantly enhance the performance of optical microscopy in terms of resolution and sensitivity through a thorough analysis of the statistical features of input signals [19,20,21]. Recently, an ANN has been applied to a MIR gas spectrometer to accelerate the estimation of gas concentration in complex gas matrix conditions, with it showing promising results [22,23]. In this paper, we propose to use a multilayer perceptron (MLP) to provide an accurate prediction of the gas concentration from the measured gas absorption spectra that suffer from a random spectral shift that is induced by inevitable mechanical stress imposed onto optical components of our MIR trace gas analyzer, explicitly providing quantitative analysis based on the experimental data.

## 2. Development of the MIR Gas Spectrometer and the Spectral Shift Issue

**Figure 1:**
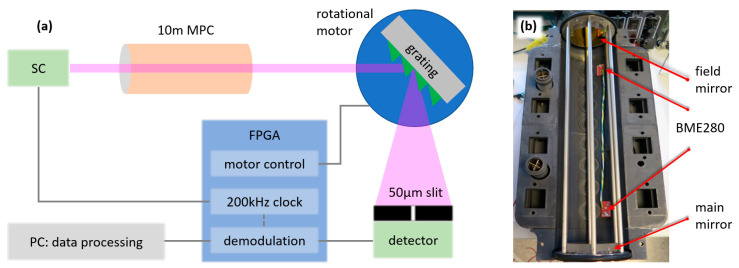
(**a**) Simplified schematic diagram of the scanning grating-based MIR SC gas spectrometer. (**b**) Photo of the multipass gas cell (MPC) with a standard White cell optical configuration. BME280 is an electrical sensor to monitor temperature, humidity, and pressure inside the MPC.

Figure 1a depicts the simplified schematic diagram of the scanning grating-based MIR trace gas sensing system that we have developed in our laboratory, using a supercontinuum (SC) light source that is spectrally broadened from 2 µm to ~4.5 µm. The SC light is sent to a multipass cell (MPC), which consists of two mirrors with a standard White cell optical configuration, as shown in Figure 1b. The mirrors are glued on a metal frame at fixed positions to predetermine the total optical path length of 10 m. Consequently, the interaction length between light and gas analytes becomes extensively increased through a compact design, and the wavelength-specific absorption is accordingly enhanced, resulting in a considerable improvement in the sensing sensitivity. Then, the light emerging from the cell is directed to a blazed grating with 450 lines/mm. The first-order diffracted light was focused on a single-pixel detector for the gas absorption analysis. The spectrum of the SC light was readily resolved by rotating the grating that is mounted on a motorized rotational stage. When using a free space beam path length of 36 cm between the grating and the detector and a grating dispersion of 27.9 µm/°, a grating rotation increment of 0.01° induced a rise in the geometrical beam steering of 60 µm. This corresponds to a spectral shift of 0.51 cm^−1^ in wavenumber. Based on these parameters, a 50 µm slit was placed in front of the detector to precisely record the spectral power density of the light source, resulting in a spectral resolution of 1.15 cm^−1^ for the sensing system. The grating was then scanned from 327° to 330° by steps of 0.01° to obtain the partial absorption spectrum of water vapor contained in the ambient air while the ambient air was pumped into the cell. Notice that the free space path length from the light source to the detector is ~90 cm, excluding the beam path through the cell, hence proving that the light absorption inside the cell would be dominant compared to the light absorption occurring in the open space portions.

**Figure 2:**
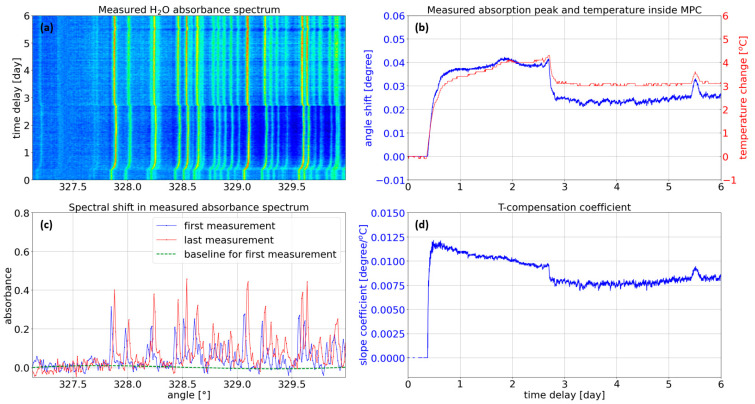
(**a**) Continuous measurement of H_2_O absorption profile while the gas cell temperature changes. (**b**) Slope coefficient calculated by the ratio of the amount of spectral shift to temperature change. (**c**) H_2_O absorbance spectra for the first and last measurements, showing a clear spectral shift due to the temperature change inside the MPC. (**d**) Measured slope coefficient defined as the ratio of the spectral shift to temperature change.

From the measured H_2_O absorbance spectra, as shown in Figure 2a, a spectral shift of water absorption peaks over time is clearly observed. The amount of the spectral shift was then compared to the change of temperature inside the MPC, showing a strong correlation, as shown in Figure 2b. The temperature inside the MPC, as well as the humidity and pressure, was simultaneously measured by an electrical sensor (BME280, BOSCH) embedded inside the MPC. For this reason, we believe that the spectral shift can be mainly attributed to the variation of temperature inside the MPC. We suspect that the thermally induced movements of the optical components in the system lead to such detrimental beam steering, causing the spectral shift in the measurement. However, we did not investigate further to determine the cause of the beam discrepancies.

According to our measurements, the spectral shift of the absorption spectrum seems to have a complex response to the temperature change since the slope efficiency defined as the ratio of the spectral shift to temperature change varies, as shown in Figure 2d. However, the coefficient was estimated to be in the order of 0.01 °/K, implying that the measured gas absorption profile will be spectrally shifted with a coefficient of 0.51 cm^−1^/K. Figure 2c illustrates the water absorbance spectra of the first and last measurements, explicitly showing a spectral shift by 0.03° while the temperature was changed by 2.6 °C between these two measurements. On the other hand, the measured absorbance spectrum was compared to the HITRAN reference absorbance to calculate the amount of the spectral shift, resulting in a spectral shift of ~1.55 cm^−1^, showing good agreement with our estimation of the spectral shift of 0.51 cm^−1^ by grating rotation of 0.01°. Furthermore, when we performed the typical least-squares fitting algorithm to retrieve the gas concentration, we obtained 11,125 ppm (or 1.113%) and −1435 ppm (or −0.144%) for the first and last measurements, respectively. Therefore, the sensing system turned out to be severely impaired since the thermally induced spectral shift invalidated the predefined background transmission profile and the reference gas absorption profile used for the fitting algorithm. Moreover, due to the complex response of spectral shift to the temperature change, a fixed slope coefficient cannot precisely compensate for the adverse thermal effect for practical applications. More details will be discussed later.

## 3. Results and Discussion on the Proposed Artificial Neural Networks

### 3.1. Development of Artificial Neural Networks

To effectively overcome this problem, an MLP was applied to accurately estimate the gas concentration in the presence of such an inevitable spectral shift. Moreover, to validate the proof-of-concept, we decided to focus on the measurement of water vapor concentration since a capacitive relative humidity (RH) sensor embedded inside the multipass cell could be used as a reference. Due to the limited number of available measured absorbance spectra, our neural network was trained with synthetic absorbance spectra. First, we extracted the molecular absorption coefficients for water vapor (H_2_O) and methane (CH_4_) with a Lorentzian profile from the HITRAN database. As the goal was to accurately estimate the H_2_O concentration, CH_4_ was used as a perturbation since the atmospheric methane absorption is certainly present in the set scanning spectral range. The next step to generate an absorbance spectrum was to sample a CH_4_ concentration between 0 and 100 ppm and a H_2_O concentration between 0 and 10% from a scaled beta distribution with parameters a = 0.5 and b = 1. In fact, the water vapor concentration of 10% corresponds to the 100% relative humidity under environmental conditions of 46 °C and atmospheric pressure, which considers harsh environments such as gaseous pollutants monitoring generated from the incineration plant. The distribution parameters were selected to favor lower gas concentrations where estimation errors should be lower. In turn, these sampled concentrations were utilized to generate a transmittance spectrum with a 10 m optical path length, a convolution with a Gaussian kernel, and a spectral resolution of 1.15 cm^−1^. Finally, an absorbance spectrum was computed by taking the logarithm of the transmittance spectrum. Each synthetic absorbance spectrum spans the band ranging from 3010 cm^−1^ to 3290 cm^−1^ in 0.05 cm^−1^ steps and includes 5601 samples.

To mimic the practical signal noise and nonlinear spectral behavior, we applied three perturbations to the generated synthetic spectrum. Firstly, using our measurements, the level of root-mean-squared noise within a finite spectral window, at which the water vapor absorption is negligible, was analyzed. More specifically, the window in the vicinity of the grating rotation angle of 327.5°, corresponding to 3159.4 cm^−1^ in wavenumber, as shown in Figure 2c, was selected for our analysis. As a result, an additive white Gaussian noise with a standard deviation of 0.025 absorbance units was imposed onto the synthetic absorbance spectrum. Secondly, a random baseline generated as Legendre polynomials of degree 4 with coefficients sampled uniformly was also added since SC light sources typically suffer from intrinsic peak-to-peak random fluctuations in their intensity and spectral power density. Such an adverse noise is mainly attributed to the mechanism of the incoherent nonlinear spectral broadening process during supercontinuum generation [24], and it varies the baseline of the measured absorbance spectrum, as shown in Figure 2c, which leads to a non-negligible amount of error in the computed gas concentration. After analyzing the temporal variation of the baseline over the whole measurements, the range of each coefficient of the fourth-order polynomial function was thoroughly determined to generate a random baseline. Thirdly, we added a random spectral shift that is sampled uniformly between −10 cm^−1^ and 10 cm^−1^, covering the temperature change of ±19.6 °C. The last step of synthetic data generation was to scale the absorbance spectra between 0 and 1 to facilitate training. The scaling factor was determined from the maximum possible concentrations of CH_4_ and H_2_O without any perturbation.

**Figure 3:**
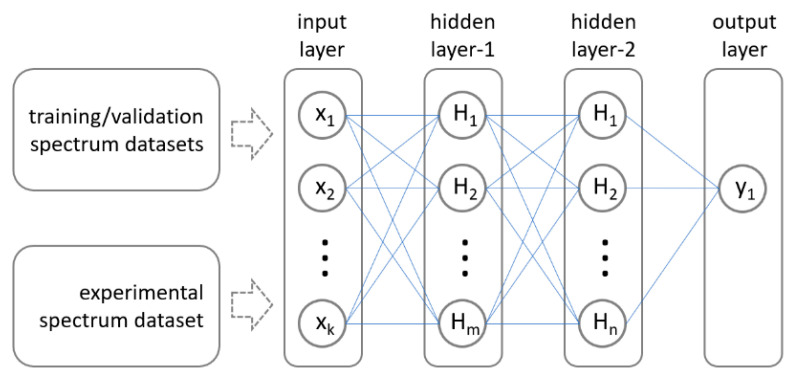
MLP architecture, consisting of input and output layers and two hidden layers with 256 units and ReLU activation.

Figure 3 illustrates the considered MLP architecture. An MLP is a fully connected feed-forward ANN, where each neuron is connected to all neurons in neighboring layers. In our work, the MLP is composed of three layers, namely, two hidden layers with 256 neurons and ReLU (Rectified Linear Unit) activation and one output layer with a single unit and linear activation. Our network takes an absorbance spectrum as input and estimates the H_2_O concentration as follows:

(1) $$\begin{array}{l} x_{1}=\mathrm{ReLU}\{W_{1}x_{0}+b_{1}\}\\ x_{2}={\mathrm{ReLU}\{W}_{2}x_{1}+b_{2}\}\\ x_{3}=W_{3}x_{2}+b_{3} \end{array}$$

where $x_{0}\in R^{5601}$ is the input spectrum; $x_{1}\in R^{256}$, $x_{2}\in R^{256}$, and $x_{3}\in R$ are the outputs of each layer; $W_{1}\in R^{256\times 5601}$, $W_{2}\in R^{256\times 256}$, and $W_{3}\in R^{1\times 256}$ are the layer weights; and $b_{1}\in R^{256}$, $b_{2}\in R^{256}$, and $b_{3}\in R$ are the layer biases. The MLP parameters were trained through back-propagation by minimizing the mean squared error (MSE) with the Adam optimizer, hence optimizing the weights and bias for each neuron. Finally, the output layer provided the result of the hidden layers as the gas concentration. The training process was performed with a learning rate of 0.001 for 500 epochs of 1000 batches, where each batch was composed of 100 synthetic absorbance spectra. This means that the MLP was presented with 50 million different spectra over the whole training procedure. At each epoch, the MLP was evaluated with a validation set of the same size as the training set. In turn, MLP parameters corresponding to the epoch with the lowest MSE on the validation set were selected for the evaluation of the real absorbance spectra.

### 3.2. Evaluation of the Proposed Artificial Neural Networks

To evaluate the reliability of the fully trained MLP algorithm, a test dataset consisting of experimentally measured absorbance spectra was applied to the network. To prepare the test dataset, we used two individual gas bottles filled with a calibration mixture of the gases of interest: one bottle of 100% nitrogen and the other of a mixed gas of 99.995% nitrogen and 0.005% (equivalent to 50 ppm) methane. The two bottles were combined before being fed into the gas cell. The gas flow rate of each bottle was controlled independently to vary the water vapor concentration between 0% and an atmospheric level of ~1% with different levels of CH_4_ perturbation. The sensing system was operated over 38 h with a measurement time of 3 min to continuously acquire the gas absorption spectra under different environmental conditions. The measurement started while the two bottles were closed and the MPC was under normal atmospheric conditions. Then, during the time window between time 14 h and 28 h, the methane concentration was set to five different levels: 0, 0.5, 1, 1.9, and 50 ppm, and each concentration remained constant over 1–2 h.

**Figure 4:**
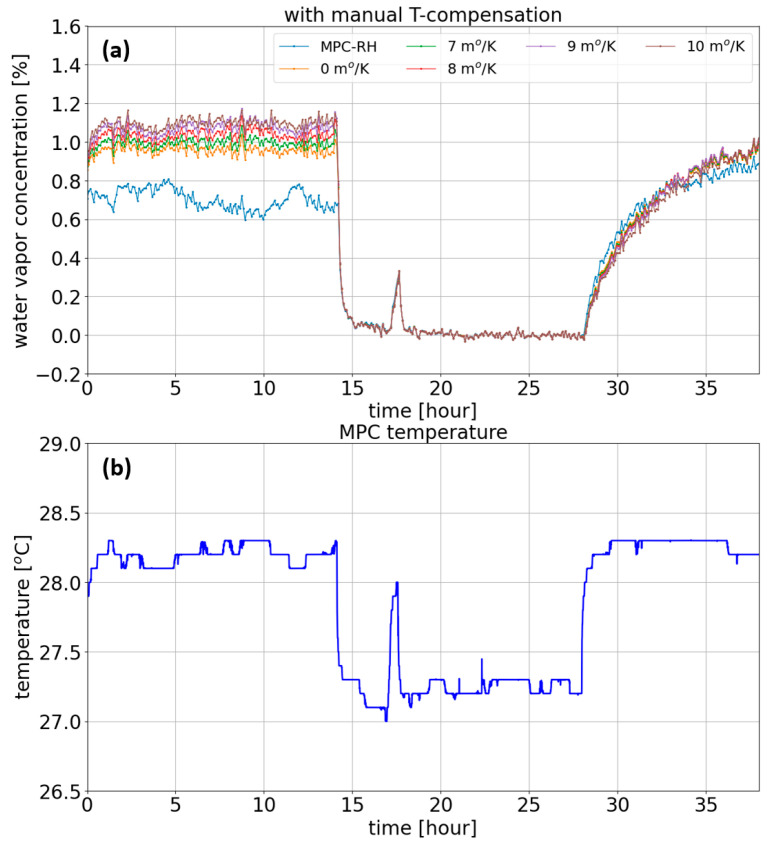
(**a**) Reference H_2_O concentration profile in black and retrieved profiles resulting from the manual temperature compensation for different coefficients. (**b**) Measured temperature inside the MPC.

The water vapor concentration measured by the relative humidity shows an atmospheric concentration of 1.05%, as shown in Figure 4a, which corresponds to 27.75% RH. However, when the MPC was fed by the sample gas, the water concentration was abruptly decreased to 0% due to the nitrogen purging effect in the gas cell. Next, the gas flow was stopped after 28 h, and the atmospheric water vapor started to enter the gas cell, increasing to about 1.1% at a measurement time of 38 h. It is important to mention that during this test period, the temperature inside the gas cell varied from 27.00 °C to 28.30 °C, as shown in Figure 4b, while the frequency calibration of the grating angle was performed at 27.25 °C. Therefore, we expect that our sensing system would be impaired by the grating angle deviation of ~0.015°, equivalent to the spectral shift of ~0.76 cm^−1^ in wavenumber. It is noticeable that the water concentration profile shows a perfect match with the temperature variation profile of the gas cell. Overall, as expected, due to the adverse spectral shift, the water concentration retrieved by the typical least-mean-squares fitting algorithm shows a 40.3% error for the atmospheric air sample at the beginning of the test (referring to the profile in blue (0 m°/K) in Figure 4a). The apparent spike in the measured water vapor concentration and the temperature of the gas cell in Figure 4 was, unfortunately, caused by the replacement of the nitrogen gas bottle since the bottle became empty. During replacement, penetration of ambient air into the gas cell was inevitable.

To evaluate the performance of the manual temperature compensation, the original absorption spectrum was spectrally corrected using five different slope coefficients at 0.007–0.011°/K, as shown in Figure 4a. The computed water vapor concentration with the manual temperature compensation becomes closer to the reference RH profile as the slope efficiency increases, resulting in a large computation error of −2.8% and −40.3% for slope efficiencies of 0.011°/K and 0°/K, respectively.

**Figure 5:**
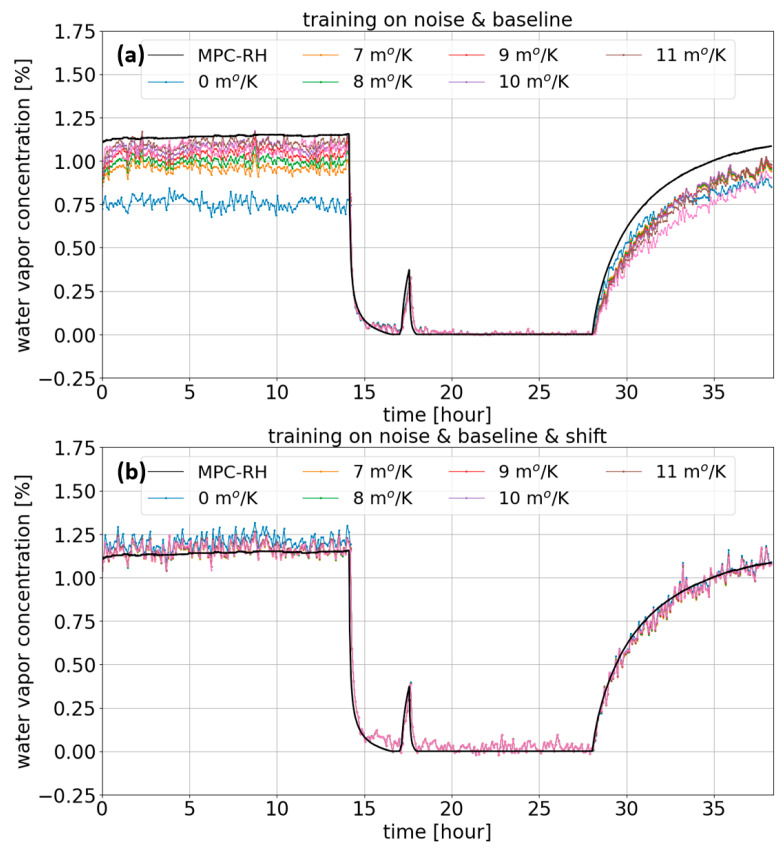
H_2_O concentration estimated by the ANN (**a**) when MLP training relied on the noise and baseline and (**b**) when MLP training relied on the noise, baseline, and spectral shift.

Then, all gas absorption spectra, including the original ones and those spectrally shifted by the manual temperature compensation, were applied to the trained network. To explicitly evaluate the importance of training on the spectral shift, we developed two MLP network algorithms. The first network was trained on two features of the system noise and the baseline of the light transmission spectral profile with the random parameter range, as previously explained. But, as shown in Figure 5a, the trained network demonstrated nearly the same results as the typical fitting result, leading to a large error in the computed gas concentration. This means that the capacity of this network was insufficient to overcome the complex spectral shift problem. On the contrary, when our second MLP network was trained, we included the feature of the temperature-induced spectral shift. It is remarkable that the water vapor concentration predicted by the second network turned out to be considerably precise, as shown in Figure 5b. All temperature-compensated spectra with a different slope coefficient converged to an identical pattern of concentration variation in time. Moreover, this results in good agreement with the reference profile, showing a marginal error of less than ±1% while preserving the sensing sensitivity, which was determined by the standard deviation of the concentration fluctuation over the first 12 h measurements. Therefore, this proves the large potential of completely overcoming such unpredictable spectral shifts induced in light absorption-based gas spectrometers. In addition, we believe that the performance of the ANNs for this task could be further improved by investigating more complex architectures, including convolutional and pooling layers.

## 4. Conclusions

In conclusion, we have identified a temperature-induced spectral shift issue in our scanning grating-based MIR gas sensing system and have proposed a promising solution to deal with such a detrimental thermal effect. According to our successful experimental demonstration, an ANN is a promising approach to unambiguously overcome any presence of complex spectral distortion imposed on the spectroscopic data. Due to the adverse global spectral shift imposed onto the measured gas absorption spectrum, the conventional least-mean-squares fitting algorithm suffers from a severe error in the gas concentration computation, leading to a severe error as large as 40.3%, which cannot be effectively improved by a manual temperature compensation method due to the nonlinear response. However, our proposed MLP algorithm which statistically relates the distorted spectrum to the pure reference spectrum enables us to significantly improve the accuracy of the retrieved gas concentration with a marginal error of less than ±1%.

The degradation of the sensing performance by such a spectral shift can occur in any spectroscopic sensing system that requires an accurate calibration of frequency in the measurement. So, we can emphasize that the proposed solution has a large potential to make trace gas sensing systems based on the gas absorption spectrum more robust and immune to any variation of environmental conditions. In addition, we believe that machine learning will also be powerful when applied to any type of spectral distortion once they have been properly characterized.

## Data Availability

Not applicable.
